# Comparing thermal imaging and non-contact infrared thermometers for monitoring skin temperature in a prospective cohort with lower limb cellulitis

**DOI:** 10.1136/bmjopen-2025-100667

**Published:** 2025-07-18

**Authors:** Elizabeth L A Cross, Martin John Llewelyn, (Ann) Sarah Walker, Gail Hayward

**Affiliations:** 1 Department of Global Health and Infection, Brighton and Sussex Medical School, Brighton, UK; 2 Department of Infection, University Hospitals Sussex NHS Foundation Trust, Brighton, UK; 3 Nuffield Department of Medicine, University of Oxford, Oxford, UK; 4 NIHR Oxford Biomedical Research Centre, Oxford, UK; 5 National Institute for Health Research Health Protection Research Unit in Healthcare Associated Infections and Antimicrobial Resistance, Oxford, UK; 6 Nuffield Department of Primary Care Health Sciences, University of Oxford, Oxford, UK; 7 Healthtech Research Centre in Community Healthcare, National Institute for Health and Care Research, Oxford, UK

**Keywords:** Infectious diseases & infestations, INFECTIOUS DISEASES, Dermatology

## Abstract

**Abstract:**

**Objective:**

Skin temperature assessment is essential for the diagnosis of cellulitis and monitoring treatment response, but is currently subjective and can contribute to overdiagnosis. We aimed to characterise skin temperature changes over time in cellulitis and compare two objective measurement approaches, a thermal imaging camera (TIC) and a non-contact infrared thermometer (NCIT).

**Design:**

A device comparison study nested within a prospective cohort. We measured limb temperatures daily for 4 days using a TIC and two NCITs.

**Setting:**

Two acute hospitals in the UK’s National Health Service.

**Participants:**

202 adults (age ≥18 years) diagnosed with lower limb cellulitis who attended hospital for antibiotic treatment.

**Outcome measures:**

We used linear mixed-effects models to quantify changes in temperature over time and intraclass correlation coefficients (ICC) to assess reliability. We compared temperature measurements between devices using Lin’s concordance coefficients and Bland-Altman plots with estimated 95% limits of agreement.

**Results:**

202 patients were included: 95% white ethnicity. Baseline limb temperature differences varied between 2.4°C and 3.4°C, depending on the device. All devices showed significant reductions in affected limb temperature per day, with the largest decrease recorded by the TIC (−0.34°C per day, 95% CI −0.48°C to −0.19°C, p<0.001). Only the TIC and NCIT-1 showed significant reductions in limb temperature difference per day. All devices had excellent reliability (ICCs ≥0.98). However, the magnitude of daily temperature change estimates was small relative to the repeatability coefficients of each device. The NCIT-2 consistently recorded the smallest differences in limb temperatures and demonstrated evidence of proportional bias.

**Conclusions:**

Daily temperature changes may be too small for reliable monitoring at the individual patient level, but cumulative changes from day 0 to day 3 may be sufficient for clinical interpretation, despite limitations in the precision of device measurements. NCITs’ measurement capabilities differ widely, so these devices cannot be used interchangeably. Due to this and the potential benefits of advanced thermal image analysis, TICs should be prioritised for further study in cellulitis. Future research should confirm our findings in different skin tones and explore the clinical utility of thermal imaging in enabling earlier diagnosis or detecting signs of therapeutic failure.

**STRENGTHS AND LIMITATIONS OF THIS STUDY**

- Our study is the first to directly compare two emerging technological approaches for objective skin temperature monitoring in cellulitis.
- Limb temperature progression was measured daily, providing insights into early clinical response in patients with more severe cellulitis than in previous studies.
- The reliability and repeatability of skin temperature measurements, which have not been previously explored, were assessed and compared across devices.
- Measurements were recorded using feasible and practical methods, making them applicable to everyday clinical settings.
- Nearly all study participants were of white ethnicity, so findings must be confirmed for patients with different skin tones.

## Introduction

 Cellulitis is a common bacterial skin infection characterised by warmth, pain, swelling and acute colour change of the affected skin.^1^ Skin temperature assessment is essential for both the initial diagnosis of cellulitis (to differentiate from mimics such as varicose eczema and lipodermatosclerosis) and for monitoring response to antibiotic treatment.^2 3^ In current practice, this is a subjective clinical assessment and is likely to be very unreliable, especially when conducted by different clinicians over time. One study showed that even substantial temperature differences in extremities of >3°C were only detected by clinicians 76% of the time.^4^ Unsurprisingly, therefore, cellulitis is both overdiagnosed^5 6^ and overtreated, with 30%–50% of patients experiencing unnecessarily prolonged antibiotic treatment.^7^,^9^

Technological solutions that provide an objective assessment of skin temperature can potentially improve diagnostic accuracy in cellulitis, thus improving patient outcomes, reducing unnecessary antibiotic treatment and associated harms, including antibiotic resistance and reducing healthcare costs. Two broad approaches have been applied: non-contact infrared thermometers (NCITs)^10^,^12^ and thermal imaging cameras (TICs).^13^,^18^ Diagnostic studies using these devices in cellulitis have found significant temperature differences between affected and unaffected limbs.^13^,^1618^ However, few studies have monitored temperatures beyond the point of diagnosis,^10 12 17^ and none have attempted to compare these two technologies. Therefore, the objective of our study was to characterise skin temperature changes over time in cellulitis and compare these two approaches.

## Methods

### Patient and public involvement

This study involved patients and the public in the design and conduct of the research through the James Lind Alliance Cellulitis Priority Setting Partnership^19^ and a patient and public involvement (PPI) group consisting of people with lived experience of cellulitis. Our PPI contributors were involved from the study design stage; they helped to check the acceptability of the study procedures, edited patient information materials and improved the definitions and collections of outcome measures.

### Study design and population

This device comparison study of two technological approaches (one TIC and two NCITs) was nested within a prospective cohort study of patients with cellulitis conducted between June 2021 and March 2023.^20^ The study was conducted at two acute hospitals in the UK’s National Health Service (NHS): a large tertiary referral hospital and a district general hospital, both within University Hospitals Sussex NHS Foundation Trust.

Adults (aged ≥18 years) were eligible if their treating clinician identified them as having lower limb cellulitis that required antibiotic treatment. The main exclusion criteria were having received three or more calendar days of antibiotics from the hospital for cellulitis prior to study enrolment or having been treated for a previous episode in the preceding 28 days. Patients were also excluded if the clinical diagnosis changed to an alternative diagnosis within 3 days of enrolment or if the patient, in the judgement of the investigator, did not have a clear diagnosis of cellulitis, to enable the exclusion of infections, such as severe/deep diabetic foot infections, which may be loosely labelled as cellulitis but are treated with different guideline antibiotic agents and durations. Further exclusion criteria are detailed in online supplemental materials p2.

### Devices

Two devices were evaluated throughout the whole study.

TIC) FLIR ONE Gen 3—Android USB-C (Teledyne FLIR, USA), a TIC that attaches to a smartphone with an object temperature range of −20°C to +120°C and a reported accuracy of ±3°C.

NCIT-1) Extech IR200 (Extech Instruments Corporation, USA), an NCIT with a surface temperature range of 0°C–60°C and reported accuracy ±0.8°C.

A third device became available in the study at month 9 and was used on 103 (51%) study patients.

NCIT-2) Thermofocus 0800 /H5 (Tecnimed s.r.l., Italy), an NCIT with a measuring range of 1.0°C to 55.0°C and a reported accuracy of ±0.2°C to ±1.0°C, dependent on the measuring temperature and least accurate at extremes of range.

### Procedures

Temperature measurements were taken at the point of maximal temperature on the affected limb and at the corresponding point on the non-affected limb to allow calculation of temperature difference (affected minus unaffected limb temperature) (details in online supplemental materials p2).

To calculate reliability, repeated measurements were taken from both the affected and unaffected limbs (two measurements for the TIC and three for the NCITs, because a priori it was hypothesised that measurements from the NCITs would be more variable, and taking another repeat measurement added negligible extra time for these devices (<10 s) in contrast to the ~2 min for each TIC reading and image upload). Temperature readings were made approximately 10 min after removing any clothes or dressings. The devices were held at room temperature for at least 10 min before readings were taken. Measurements were taken indoors in temperature-regulated clinical areas. Temperature measurements were not provided to treating clinicians.

Where possible, temperature measurements were performed on all patients daily for 4 days beginning on day 0, defined for the study as the date the patient began their hospital-associated antibiotic treatment for cellulitis (61 (30%) were already taking antibiotics prescribed in the community for a median 3 days (IQR 2, 4), in which case day 0 was when the prescription was changed in hospital). Where patients were enrolled after day 0, temperature readings were only available from enrolment. Where patients were discharged before day 3, readings were only available until discharge.

### Statistical analysis

#### Skin temperature over time

For each device, linear mixed-effects models were used to quantify the mean day 0 temperature and daily change in affected, unaffected and limb temperature difference, with correlated participant-level random effects for baseline and daily change. Conditional on these random effects, repeated measurements taken within each participant on each specific day were considered independent.

#### Device comparison

To assess reliability, intraclass correlation coefficients (a reliability index that measures the degree of correlation and agreement between measurements) were calculated using a one-way random-effects model to assess the absolute agreement of repeated measurements. Repeatability, defined as the consistency of measurements when taken repeatedly at short intervals by the same device under the same conditions, was assessed by calculating the repeatability coefficient using the ‘REPEATABILITY’ module in Stata,^21^ estimating 95% CIs from 1000 bootstrap samples. The repeatability coefficient can be interpreted as the range (between the negative and positive values of the coefficient) within which the difference between any two repeated measurements on the same subject is expected to lie for 95% of subjects.^22 23^ Due to the late introduction of NCIT-2 into the study, we performed a sensitivity analysis comparing the repeatability coefficients over the same time period for the TIC and NCIT-1 when NCIT-2 was in use.

Lin’s concordance correlation coefficient was calculated to determine the agreement on temperature obtained by the devices.^24^ The value increases as a function of the nearness of the data’s reduced major axis to the line of perfect concordance (the accuracy of the data) and of the tightness of the data about its reduced major axis (the precision of the data).

The difference in the mean of each patient’s skin temperature measurement from each pair of devices was plotted against the mean of these two mean measurements to create a Bland-Altman plot,^25^ and the 95% limits of agreement (LOA) were estimated.

Analyses were conducted using mean values of repeated measurements for a participant at a specific time point, apart from calculations relating to modelling skin temperature change over time, reliability and repeatability, where the original repeated measurements were used. Outlying repeated measurements were removed based on the frequency distributions of the SD of repeated temperature measurements (online supplemental figures 1 and 2).

#### Sample size

The sample size for the cohort study (N=220, allowing for 10% lost to follow-up) was determined by its primary objective to identify predictors of cellulitis recurrence.^20^ This was, therefore, the limit on this method’s comparison study. Stata V.18.0 software (StataCorp) was used for all statistical analyses.

## Results

202 patients were included; the median age was 66 years (IQR 51, 79), 84 (42%) were female and 191 (95%) were of white ethnicity.

For the TIC and NCIT-1, across days 1–3, missing data ranged from 16% to 29%, whereas day 0 data were missing for 69%, reflecting enrolment after initiation of hospital antibiotics (day 0) in the majority (online supplemental table 1). As NCIT-2 measurements were performed on fewer patients, missing data were 59%–65% and 80%, respectively.

### Skin temperature over time

#### Absolute limb temperature

**Figure 1:**
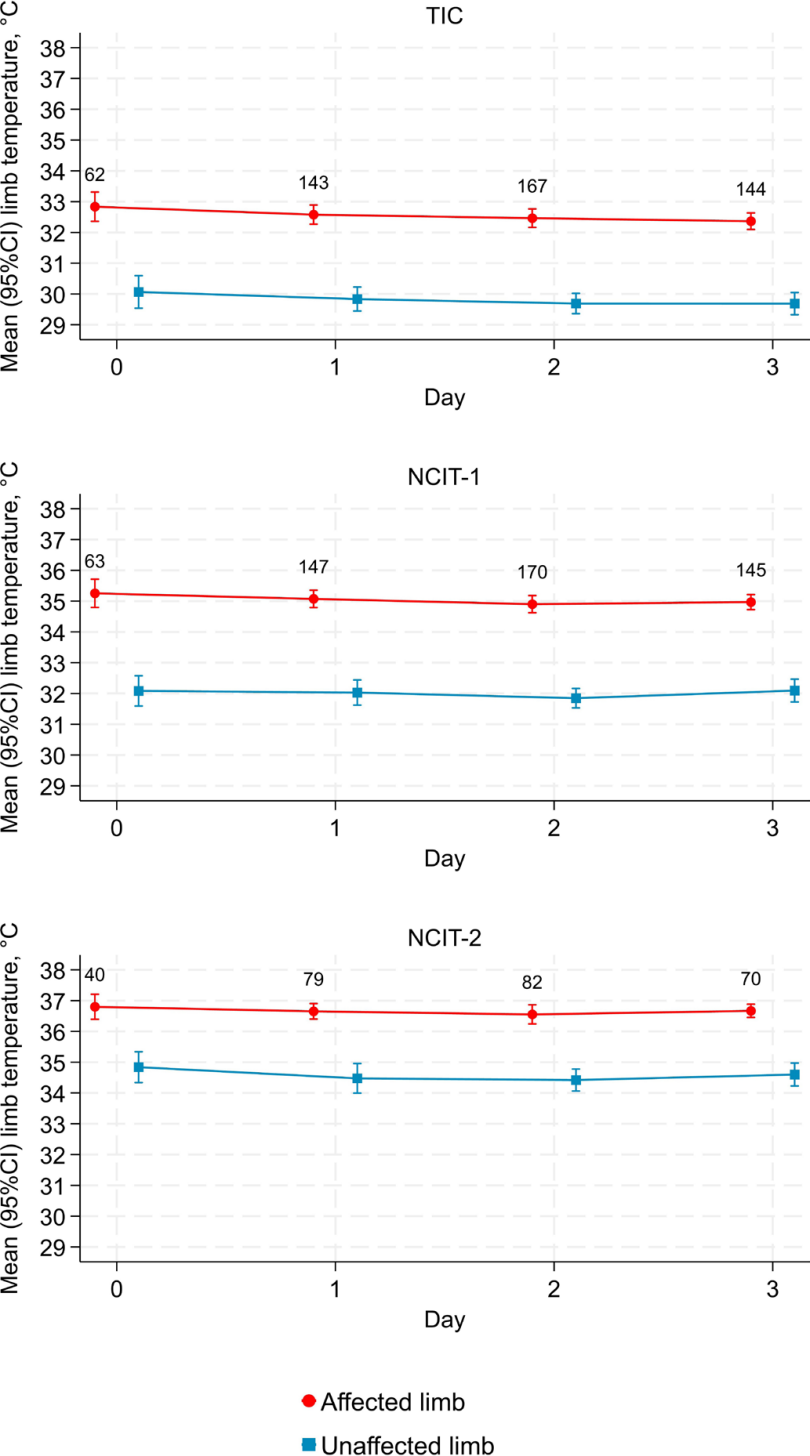
Mean (95% CIs) temperature of affected and unaffected limbs over days 0–3. Day 0 was taken as the date of hospital antibiotic initiation. Numbers show the number of participants with data (out of a total of 202 with any data for TIC and NCIT-1 and 103 for NCIT-2). NCIT, non-contact infrared thermometer; TIC, thermal imaging camera.

Across days 0–3 and for all devices, the mean affected limb temperature was warmer than the mean unaffected limb temperature (figure 1). Including all repeated measurements in linear mixed models, the estimated day 0 affected limb temperatures were 33.06°C (95% CI 32.68°C to 33.44°C) for the TIC, 35.22°C (34.83°C to 35.61°C) for NCIT-1 and 36.89°C (36.56°C to 37.20°C) for NCIT-2 (table 1).

**Table 1:** Mean daily change in temperature in affected and unaffected limbs, and mean temperature difference between affected and unaffected limbs

Measurement	Device	Mean estimated temperature day 0	95% CI	Mean change per day (°C)	95% CI	P value	Correlation between baseline and daily change	95% CI	N
Affected limb temperature	TIC	33.06	32.68 to 33.44	−0.34	−0.48 to −0.19	<0.001	−0.70	−0.78 to −0.58	1029
	NCIT-1	35.22	34.83 to 35.61	−0.20	−0.37 to −0.03	0.02	−0.79	−0.84 to −0.71	1567
	NCIT-2	36.89	36.56 to 37.20	−0.20	−0.38 to −0.02	0.03	−0.65	−0.76 to −0.50	811
Unaffected limb temperature	TIC	29.95	29.48 to 30.42	−0.11	−0.29 to 0.07	0.24	−0.79	−0.85 to −0.71	1029
	NCIT-1	31.85	31.37 to 32.32	0.05	−0.15 to 0.24	0.63	−0.81	−0.86 to −0.75	1563
	NCIT-2	34.48	34.00 to 34.97	0.02	−0.20 to 0.23	0.88	−0.74	−0.83 to −0.62	807
Limb temperature difference	TIC	3.10	2.75 to 3.44	−0.22	−0.37 to −0.07	0.004	−0.78	−0.84 to −0.71	1029
	NCIT-1	3.35	2.91 to 3.80	−0.24	−0.44 to −0.04	0.02	−0.80	−0.85 to −0.73	1558
	NCIT-2	2.39	1.85 to 2.92	−0.20	−0.49 to 0.09	0.18	−0.79	−0.86 to −0.70	805

From linear mixed models.

NCIT-1, non-contact infrared thermometer; TIC, thermal imaging camera.

The temperature in the affected leg decreased day by day for all devices, with the largest decrease for the TIC (−0.34°C per day, 95% CI −0.48°C to −0.19°C, p<0.001) (table 1). There was no evidence of a change in temperature of the unaffected leg per day for any device (p>0.2). In the affected leg, baseline temperature and change per day were strongly negatively correlated for all devices, that is, limb temperatures declined the fastest in patients who started with higher limb temperatures.

#### Limb temperature difference

**Figure 2:**
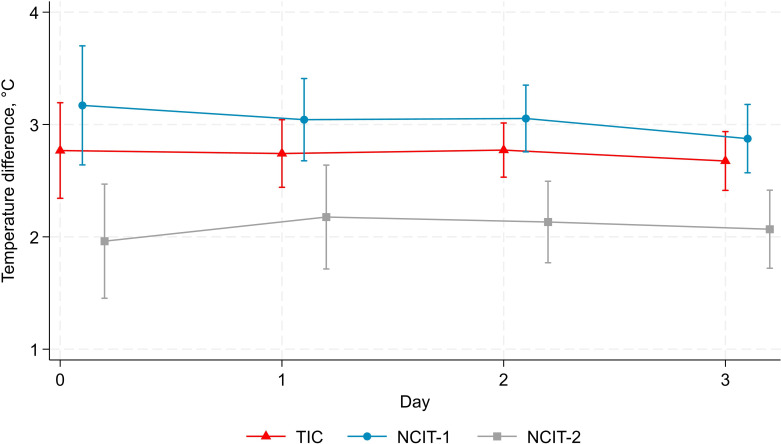
Mean (95%CIs) limb temperature difference over days 0–3. NCIT, non-contact infrared thermometer; TIC, thermal imaging camera.

Across days 0–3, the largest mean temperature differences were recorded by NCIT-1 and the smallest by NCIT-2 (figure 2). Including all repeated measurements in linear mixed models, the estimated day 0 limb temperature differences were 3.10°C (95% CI 2.75°C to 3.44°C) for the TIC, 3.35°C (2.91°C to 3.80°C) for NCIT-1 and 2.39°C (1.85°C to 2.92°C) for NCIT-2 (table 1).

The limb temperature difference decreased day by day only for the TIC and NCIT-1 (table 1), by −0.22°C (95% CI −0.37°C to −0.07°C, p=0.004) and −0.24°C (95% CI −0.44°C to −0.04°C, p=0.02), respectively. Again, baseline temperature difference and change per day were strongly negatively correlated, that is, limb temperature differences declined the fastest in patients who started with the greatest limb temperature differences.

### Device comparison

#### Reliability and repeatability

All three devices had excellent reliability with one-way random effects, absolute agreement, single rater intraclass correlation coefficients for repeated affected and unaffected limb temperature measurements of ≥0.98 (online supplemental table 2).

Repeatability varied significantly between devices and was consistently better for affected limb measurements (online supplemental table 2). Repeatability was best for NCIT-2 (0.34°C 95% CI 0.30°C to 0.37°C), worse for NCIT-1 (0.54°C (0.50°C to 0.58°C) and worst for the TIC (0.68°C (0.61°C to 0.75°C) in the affected limb measurements. A sensitivity analysis restricted to the study period when all three devices were in use produced comparable results (online supplemental table 2).

#### Agreement for affected limb temperature

**Figure 3:**
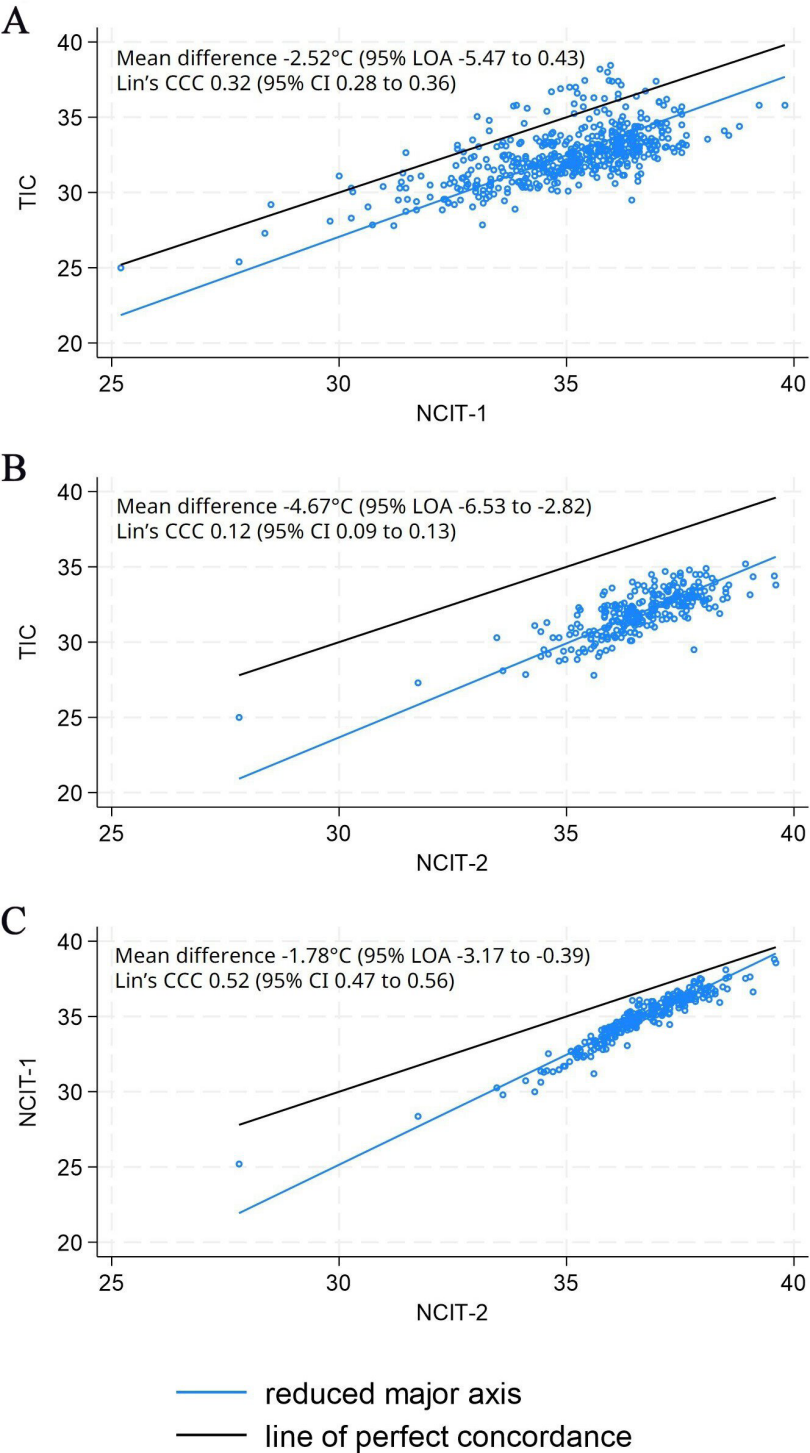
Comparison of measurements of affected limb temperature (A) TIC versus NCIT-1 (**b**) TIC versus NCIT-2 (**c**) NCIT-1 versus NCIT-2. A mean difference of −2.52°C (95% LOA −5.47 to 0.43) for the TIC versus NCIT-1 means that, on average, the TIC measures 2.52°C lower than NCIT-1 and that 95% of the measurement differences between devices will be between −5.47°C and 0.43°C. CCC, concordance correlation coefficient; LOA, limits of agreement; NCIT, non-contact infrared thermometer; TIC, thermal imaging camera.

The three devices recorded markedly different temperatures. The TIC recorded, on average, temperatures that were lower than NCIT-1 and NCIT-2, by −2.52°C (95% LOA −5.47°C to 0.43°C) and −4.67°C (95% LOA −6.53°C to −2.82°C), respectively (figure 3, online supplemental figure 3). The largest mean differences and the lowest Lin’s concordance coefficient were observed between the TIC and NCIT-2.

When comparing the TIC and NCIT-2 (and NCIT-1 vs NCIT-2), the methods did not agree equally through the range of temperature measurements; as the mean temperature decreased, the difference between the measurements increased, indicating proportional bias (figure 3, online supplemental figure 3). No such trend was observed comparing the TIC to NCIT-1, suggesting that NCIT-2 might overestimate to a greater extent at lower temperatures.

#### Agreement for limb temperature difference

**Figure 4:**
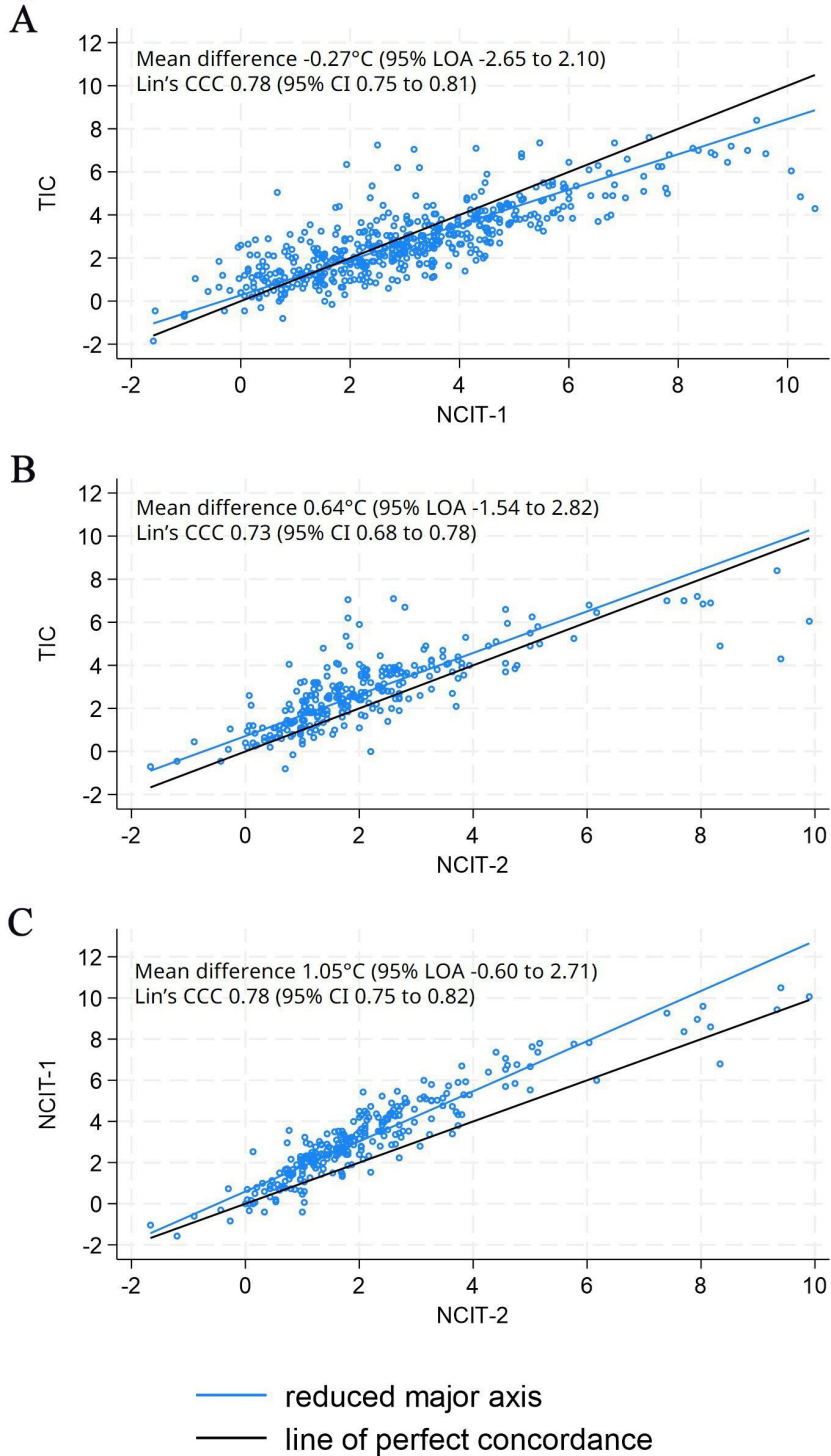
Comparison of measurements of limb temperature difference (**a**) TIC versus NCIT-1 (**b**) TIC versus NCIT-2 (**c**) NCIT-1 versus NCIT-2. CCC, concordance correlation coefficient; LOA, limits of agreement; NCIT, non-contact infrared thermometer; TIC, thermal imaging camera.

There was greater agreement (higher Lin’s concordance coefficients) for limb temperature difference than affected limb temperatures (figure 4, online supplemental figure 4). The TIC recorded, on average, lower limb temperature differences than NCIT-1 by −0.27 (95% LOA −2.65 to 2.10) and higher limb temperature differences than NCIT-2 by 0.64 (95% LOA −1.54 to 2.82). However, the mean difference was greater for the latter comparison.

Lin’s concordance coefficient was higher for the TIC versus NCIT-1 comparison, 0.78 (95% CI 0.75 to 0.81), than TIC versus NCIT-2, 0.73 (95% CI 0.68 to 0.78). As expected, the evidence of proportional bias seen for affected limb temperature between the TIC and NCIT-2 was reduced when measuring limb temperature difference, given the extent and direction of bias would be the same for both limbs.

## Discussion

In patients with lower limb cellulitis prescribed antibiotics from hospital settings, the affected limb remained hotter than the unaffected limb from day 0 to day 3. Baseline affected limb temperatures varied widely depending on the measurement device. All devices recorded a significant reduction in affected limb temperature per day, but only the TIC and NCIT-1 recorded significant reductions in limb temperature difference per day, although this could be related to lower power to detect differences using NCIT-2 due to fewer measurements. Nonetheless, NCIT-2 consistently recorded the smallest differences in limb temperatures, and there was evidence of proportional bias in this device, most likely due to NCIT-2 overestimating temperatures. Therefore, we would not recommend further investigating NCIT-2 to diagnose or monitor skin temperature in cellulitis.

The TIC had the poorest repeatability, but the fewer repeated measurements performed for this device (2 vs 3) could explain the difference. We report anecdotally that the visualised temperature of the leg with the TIC appeared to fluctuate slightly in a pulse-like manner for some patients, which was assumed to be due to the patient’s actual pulse and may also explain the poorer repeatability.

A key strength of this study is its direct comparison of two emerging technological approaches for objectively measuring limb temperature in cellulitis, helping to identify which device warrants further investigation. Additionally, daily measurements allowed us to characterise early clinical response to treatment in patients with more severe disease than those included in previous studies. A main study limitation was the high missing data at baseline (day 0), due to admissions outside of the study’s working hours. Nonetheless, our use of linear mixed models enabled the estimation of temperature decreases over time, assuming data are missing at random (which includes dependence on previous/subsequent values). Some environmental factors, such as prior patient positioning and room temperature, could not be fully controlled. However, the unaffected limb should have served as an internal control, and this reflects the constraints that would be present on a device operating in real-world clinical settings. Furthermore, our study included predominantly patients of white ethnicity, so our findings must be confirmed for patients with different skin tones.

Our use of a pragmatic case definition, ‘clinician-treated cellulitis’, means that some cases may not have had true cellulitis, as misdiagnosis rates have been reported to exceed 30%.^26^ We attempted to mitigate this by excluding patients if their clinical diagnosis changed within 3 days of enrolment or if the investigator judged they did not have a clear diagnosis of cellulitis. While this approach is less rigorous than using a blinded consensus review panel or multiple independent clinical assessments, the objective of this analysis was to evaluate device performance rather than diagnostic accuracy. Previous studies have established diagnostic thresholds for limb temperature difference in cellulitis between 0.47°C and 0.80°C^1113^,^15^; but we could not assess the actual proportion of our patients meeting these thresholds at baseline due to missing data.

Our findings suggest that TIC and NCIT-1 produce similar limb temperature difference values, suggesting they could be used interchangeably. However, the most recent diagnostic study suggested a threshold of 31.2°C in the absolute affected limb temperature (not temperature difference) achieved the highest sensitivity and negative predictive value to diagnose cellulitis.^18^ While mean baseline affected limb temperatures in our study did exceed this threshold, as measured absolute temperatures varied so markedly between devices, we would not recommend interchangeable use in this context.

Our study aligns with prior studies using NCITs to monitor limb temperature over time.^10 12^ Montalto *et al* reported a mean baseline limb temperature difference of 3.5°C (95% CI 3.0°C to 3.9°C), consistent with our TIC and NCIT-1 findings.^10^ Williams *et al* found a slightly lower median baseline difference of 2.3°C (IQR 1.2°C, 3.6°C).^12^ While not directly comparable to our data, this slightly lower value may be explained by their inclusion of patients with milder cellulitis or using a different NCIT. In terms of temperature changes over time, Williams *et al* estimated a mean reduction in affected limb temperature of 1.4°C (95% CI 1.0 to 1.8, p<0.001) in trial patients by day 5.^12^ Given we only measured through day 3, this is broadly consistent with our estimate of −0.34°C decrease per day measured by the TIC.

While our analysis focused on population-level temperature trends, the broader aim of this research is to assess the potential for temperature monitoring devices to inform individual-level treatment decisions. Our estimates of daily temperature decreases were smaller than the potential range suggested by the repeatability coefficients calculated for the devices, raising important questions about their ability to capture day-by-day changes that can guide clinical decision-making. However, some patients will have experienced larger temperature decreases than the overall mean estimates, which may still be detectable despite device imprecision. Notably, for the TIC, the mean daily change in absolute affected limb temperature was larger (−0.34°C) than for the other devices and larger than changes in limb temperature difference, suggesting that this device or absolute temperature measures may be better able to detect meaningful changes over time on an individual basis. In addition, if measurements are compared across wider time intervals, such as between day 0 and day 3, the overall change may be large enough to be clinically meaningful, despite limitations in measurement precision. Using daily averages from repeated measurements may also help mitigate this problem.

Only one study has monitored cellulitis over time using thermal imaging.^17^ A key question raised by Amendola and colleagues is whether changes in maximal temperature or changes in the size of the affected area are more important.^17^ They analysed thermal images using a fiducial marker to estimate the relative size of the affected area. They found daily reductions in severity (ie, normalised temperature) and scale (ie, affected area with elevated temperature), but the unit changes reported are difficult to interpret and cannot be compared with our findings. It is possible that more advanced image processing, used to track changes in the size of the affected area, could be a more reliable measure than monitoring changes in maximum temperature.

From our findings and due to a lack of a gold standard, there is no clear superior method between the TIC and NCIT-1. While NCITs are cheaper and require less training, their measurement capabilities differ widely, so they cannot be used interchangeably. The TIC recorded the largest daily decreases in affected limb temperature, with the highest confidence around these estimates, suggesting it may be more responsive to detecting smaller temperature changes than the NCITs. Also, advanced thermal image analysis may offer more sophisticated tracking of disease progression.^17^ TICs may also provide other advantages; patients were keen to view their daily thermal images to track the progress of their infection, and many clinicians wanted to view the regular photographs we had taken from the previous days, as their shift patterns meant most of them had never examined the patient they were reviewing. Future research should assess TICs in diverse populations and explore their role in early diagnosis and treatment response prediction, ensuring that techniques are accessible and interpretable in clinical settings in real time.

## Supplementary material

10.1136/bmjopen-2025-100667 online supplemental file 1

## Data Availability

Data are available on reasonable request.

## References

[1] Raff AB, Kroshinsky D (2016). Cellulitis: A Review. JAMA.

[2] Hirschmann JV, Raugi GJ (2012). Lower limb cellulitis and its mimics: part I. Lower limb cellulitis. J Am Acad Dermatol.

[3] Stevens DL, Bisno AL, Chambers HF (2014). Practice guidelines for the diagnosis and management of skin and soft tissue infections: 2014 update by the infectious diseases society of America. Clin Infect Dis.

[4] Tse J, Rand C, Carroll M (2016). Determining peripheral skin temperature: subjective versus objective measurements. Acta Paediatr.

[5] Levell NJ, Wingfield CG, Garioch JJ (2011). Severe lower limb cellulitis is best diagnosed by dermatologists and managed with shared care between primary and secondary care. Br J Dermatol.

[6] Cutler TS, Jannat-Khah DP, Kam B (2023). Prevalence of misdiagnosis of cellulitis: A systematic review and meta-analysis. J Hosp Med.

[7] Quirke M, Saunders J, O’Sullivan R (2016). The management of cellulitis in emergency departments: antibiotic-prescribing practices and adherence to practice guidelines in Ireland. Eur J Emerg Med.

[8] Nathwani D, Eckmann C, Lawson W (2014). Pan-European early switch/early discharge opportunities exist for hospitalized patients with methicillin-resistant Staphylococcus aureus complicated skin and soft tissue infections. Clin Microbiol Infect.

[9] Walsh TL, Chan L, Konopka CI (2016). Appropriateness of antibiotic management of uncomplicated skin and soft tissue infections in hospitalized adult patients. BMC Infect Dis.

[10] Montalto M, Davies F, Marijanovic N (2013). Skin surface temperature: a possible new outcome measure for skin and soft tissue infection. Aust Fam Physician.

[11] Demir KK, McDonald EG, de L’Étoile-Morel S (2021). Handheld infrared thermometer to evaluate cellulitis: the HI-TEC study. Clin Microbiol Infect.

[12] Williams OM, Hamilton F, Brindle R (2023). The Natural History of Antibiotic-Treated Lower Limb Cellulitis: Analysis of Data Extracted From a Multicenter Clinical Trial. Open Forum Infect Dis.

[13] Ko LN, Raff AB, Garza-Mayers AC (2018). Skin Surface Temperatures Measured by Thermal Imaging Aid in the Diagnosis of Cellulitis. J Invest Dermatol.

[14] Li DG, Dewan AK, Xia FD (2018). The ALT-70 predictive model outperforms thermal imaging for the diagnosis of lower extremity cellulitis: A prospective evaluation. J Am Acad Dermatol.

[15] Hanumakka CR, Maroju NK, Chandrashekar L (2021). Utility of infrared thermography in differentiating cellulitis from pseudocellulitis of the lower limbs-A diagnostic accuracy study. J Am Acad Dermatol.

[16] Raff AB, Ortega-Martinez A, Chand S (2021). Diffuse Reflectance Spectroscopy with Infrared Thermography for Accurate Prediction of Cellulitis. *JID Innov*.

[17] Amendola JA, Segre AM, Miller AC (2023). Using Thermal Imaging to Track Cellulitis. Open Forum Infect Dis.

[18] Pulia MS, Schwei RJ, Alexandridis R (2024). Validation of Thermal Imaging and the ALT-70 Prediction Model to Differentiate Cellulitis From Pseudocellulitis. JAMA Dermatol.

[19] James Lind Alliance Priority Setting Partnerships (2017). Cellulitis Top 10.

[20] Cross ELA, Hayward GN, Llewelyn MJ (2024). Validation of the baseline recurrence risk in cellulitis (brrisc) score and the added impact of acute clinical response. Infectious Diseases (except HIV/AIDS).

[21] Linden A (2023). Repeatability: Stata module to compute the repeatability coefficient. Statistical Software Components S459231.

[22] Barnhart HX, Barboriak DP (2009). Applications of the repeatability of quantitative imaging biomarkers: a review of statistical analysis of repeat data sets. Transl Oncol.

[23] Bland JM, Altman DG (1996). Measurement error. BMJ.

[24] Lin LI (1989). A concordance correlation coefficient to evaluate reproducibility. Biometrics.

[25] Martin Bland J, Altman D (1986). Statistical methods for assessing agreement between two methods of clinical measurement. Lancet.

[26] Weng QY, Raff AB, Cohen JM (2017). Costs and Consequences Associated With Misdiagnosed Lower Extremity Cellulitis. JAMA Dermatol.

