# Supplementary material for: Comparing thermal imaging and non-contact infrared thermometers for monitoring skin temperature in a prospective cohort with lower limb cellulitis
Source: BMJ Open. 2025 Jul 18;15(7):e100667. doi: 10.1136/bmjopen-2025-100667 (PMC12273091; doi:10.1136/bmjopen-2025-100667)
Supplement: Supplementary file 2 [file bmjopen-2025-100667.reviewer_comments.pdf]

PEER REVIEW HISTORY

BMJ Open publishes all reviews undertaken for accepted manuscripts. Reviewers are asked to complete a checklist review form and are provided with free text boxes to elaborate on their assessment. These free text comments are reproduced below.

ARTICLE DETAILS

Title (Provisional)

Comparing thermal imaging and non-contact infrared thermometers for monitoring skin temperature in a prospective cohort with lower limb cellulitis

Authors

Cross, Elizabeth LA; Llewelyn, Martin John; Walker, Ann Sarah; Hayward, Gail

---

VERSION 1 - REVIEW

**Reviewer**	**1**
**Name**	**Kroshinsky, Daniela**
**Affiliation**	**Massachusetts General Hospital, Dermatology**
**Date**	**13-May-2025**
**COI**	**None**

I commend the authors on their ability to perform this study in such a large group of patients. Their results are important and compelling and add to an existing growing literature on the subject.

In assessing the utility of these devices, the absolute temperature matters less than the ability of the device to detect change and be internally consistent/reliable/repeatable.

Given readers may not be familiar with Stata, a definition of the repeatability coefficient may be helpful.

A daily limb temperature change of -0.22 to -0.24 combined with repeatability measures that were larger than these values introduces some concerns for accuracy of interpretation over time. An explanation from the authors on this would be helpful.

Limitations include high rate of missing data for day 0 in all groups however given the study did not aim to diagnose or manage disease, the authors management of this issue is reasonable.

**Reviewer**	**2**
**Name**	**Pulia, Michael**

Affiliation **University of Wisconsin Madison School of Medicine and Public Health, Emergency Medicine**

Date **27-May-2025**

COI **None**

In this observational study, the investigators performed a comparative evaluation of three surface thermal temperature measurement devices in a cohort of patients admitted with suspected cellulitis. Strengths of the manuscript substantially outweigh the limitations and include large sample size, detailed reporting, robust statistical approach, appropriate acknowledgment of limitations, and addressing a significant gap in the literature. Specifically, the reliability testing and repeatability coefficients are important areas of focus that have not generally been presented in prior work. The only limitation that is not fully acknowledged is using the judgement of single investigator re: presence of absence of cellulitis. This is significantly less rigorous than using a blinded consensus review panel or multiple independent clinical assessments. Although this raises concern for whether the participants had cellulitis or pseudocellulitis, the objective of this analysis was device performance it the true diagnosis should not influence this. One way to improve transparency would report this cohort as patients admitted with suspected cellulitis and acknowledge that the treating clinician's assessment of this is likely incorrect in 30+% of cases (cite Weng et al JAMA Derm 2017).

Cross, Elizabeth LA; Llewelyn, Martin John; Walker, Ann Sarah; Hayward, Gail

---

VERSION 1 - AUTHOR RESPONSE

Reviewer 1

I commend the authors on their ability to conduct this study in such a large patient cohort. Their results are important and compelling and add to an existing growing literature on the subject.

Many thanks for reviewing our manuscript and for your helpful comments.

In assessing the utility of these devices, the absolute temperature matters less than the ability of the device to detect change and be internally consistent/reliable/repeatable. Given readers may not be familiar with Stata, a definition of the repeatability coefficient may be helpful.

Yes, we agree. An explanation of the repeatability coefficient was part of a footnote to supplementary table 2, but to make it clearer, we have now moved it to the main methods section (lines 217 to 228):

Repeatability, defined as the consistency of measurements when taken repeatedly at short intervals by the same device under the same conditions, was assessed by calculating the repeatability coefficient using the "REPEATABILITY" module in Stata,[21] estimating 95% confidence intervals from 1000 bootstrap samples. The repeatability coefficient can be interpreted as the range (between the negative and positive values of the coefficient) within which the difference between any two repeated measurements on the same subject is expected to lie for 95% of

subjects.[22,23]

A daily limb temperature change of -0.22 to -0.24 combined with repeatability measures that were larger than these values introduces some concerns for accuracy of interpretation over time. An explanation from the authors on this would be helpful.

This is an important point that we had not addressed, and we agree that this should be discussed further.

Our analysis quantifies population-level trends in temperature over time using linear mixed-effects models, which estimate average baseline temperatures and rates of change while accounting for participant-level variation via random effects. However, we recognise that the overarching goal of this research is to explore whether temperature monitoring devices could, in the future, support individual-level treatment decisions. We have added information to the abstract (lines 75 to 76 and lines 81 to 83) a paragraph to the discussion to acknowledge these issues (lines 396 to 419):

Abstract:
However, the magnitude of daily temperature change estimates was small relative to the repeatability coefficients of each device…

Conclusions
Daily temperature changes may be too small for reliable monitoring at the individual patient level, but cumulative changes from day 0 to day 3 may be sufficient for clinical interpretation, despite limitations in the precision of device measurements.

Discussion:
While our analysis focused on population-level temperature trends, the broader aim of this research is to assess the potential for temperature monitoring devices to inform individual-level treatment decisions. Our estimates of daily temperature decreases were smaller than the potential range suggested by the repeatability coefficients calculated for the devices, raising important questions about their ability to capture day by day changes that can guide clinical decision-making. However, some patients will have experienced larger temperature decreases than the overall mean estimates, which may still be detectable despite device imprecision. Notably, for the TIC, the mean daily change in absolute affected limb temperature was larger (-0.34°C) than for the other devices and larger than changes in limb temperature difference, suggesting that this device or absolute temperature measures may be better able to detect meaningful changes over time on an individual basis. In addition, if measurements are compared across wider time intervals, such as between day 0 and day 3, the overall change may be large enough to be clinically meaningful, despite limitations in measurement precision. Using repeated daily averages may also help mitigate this problem.

A key question raised by Amendola and colleagues is whether changes in maximal temperature or changes in the size of the affected area are more important.[17] *They analysed thermal images using a fiducial marker to estimate the relative size of the*

affected area. They found daily reductions in severity (i.e., normalised temperature) and scale (i.e., affected area with elevated temperature), but the unit changes reported are difficult to interpret and cannot be compared with our findings. It is possible that more advanced image processing used to track changes in the size of the affected area could be a more reliable measure than monitoring changes in maximum temperature.

To improve readability after these changes, we have also moved a paragraph to earlier in the discussion (lines 343 to 347).

Limitations include high rate of missing data for day 0 in all groups however given the study did not aim to diagnose or manage disease, the authors management of this issue is reasonable.

Reviewer 2
In this observational study, the investigators performed a comparative evaluation of three surface thermal temperature measurement devices in a cohort of patients admitted with suspected cellulitis. Strengths of the manuscript substantially outweigh the limitations and include large sample size, detailed reporting, robust statistical approach, appropriate acknowledgment of limitations, and addressing a significant gap in the literature. Specifically, the reliability testing and repeatability coefficients are important areas of focus that have not generally been presented in prior work. The only limitation that is not fully acknowledged is using the judgement of single investigator re: presence of absence of cellulitis. This is significantly less rigorous than using a blinded consensus review panel or multiple independent clinical assessments. Although this raises concern for whether the participants had cellulitis or pseudocellulitis, the objective of this analysis was device performance it the true diagnosis should not influence this. One way to improve transparency would report this cohort as patients admitted with suspected cellulitis and acknowledge that the treating clinician's assessment of this is likely incorrect in 30+% of cases (cite Weng et al JAMA Derm 2017).

Thank you for taking the time to review our manuscript. We agree that we could have been more rigorous with our case inclusion criteria. We have moved some information from the supplementary materials to the methods section (lines 149 to 157) to highlight the further steps we took to reduce misdiagnosis within our cohort. We have also added to the discussion section (lines 365 to 374) to address this:

Methods:
Adults (aged ≥18 years) were eligible if their treating clinician identified them as having lower limb cellulitis that required antibiotic treatment. The main exclusion criteria were having received three or more calendar days of antibiotics from the hospital for cellulitis prior to study enrolment, or having been treated for a previous episode in the preceding 28 days. Patients were also excluded if the clinical diagnosis changed to an alternative diagnosis within three days of enrolment or if the patient, in the judgement of the investigator, did not have a clear diagnosis of cellulitis, to enable the exclusion of infections, such as severe/deep diabetic foot infections, which may be loosely labelled as cellulitis but are treated with different guideline antibiotic agents and durations.

Discussion:

Our use of a pragmatic case definition, 'clinician-treated cellulitis', means that some cases may not have had true cellulitis, as misdiagnosis rates have been reported to exceed 30%.[26] We attempted to mitigate this by excluding patients if their clinical diagnosis changed within three days of enrolment or if the investigator judged they did not have a clear diagnosis of cellulitis. While this approach is less rigorous than using a blinded consensus review panel or multiple independent clinical assessments, the objective of this analysis was to evaluate device performance rather than diagnostic accuracy. Previous studies have established diagnostic thresholds for limb temperature difference in cellulitis between 0.47-0.80°C;[11,13-15] but we could not assess the actual proportion of our patients meeting these thresholds at baseline due to missing data.
